# Supplementary figures and images for: The Replisomes Remain Spatially Proximal throughout the Cell Cycle in Bacteria
Source: PLoS Genet. 2017 Jan 23;13(1):e1006582. doi: 10.1371/journal.pgen.1006582 (PMC5293282; doi:10.1371/journal.pgen.1006582)

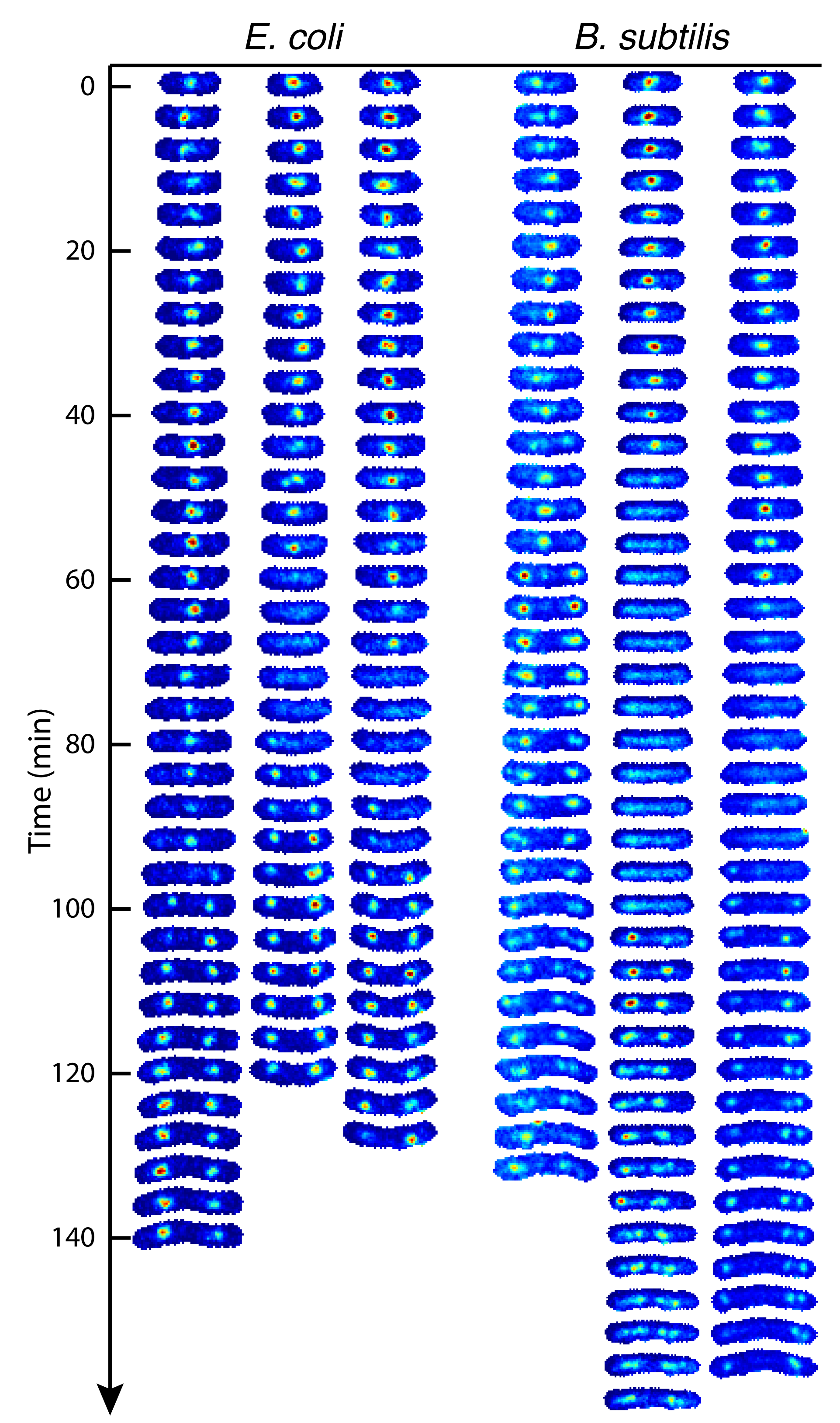

Supplement: S1 Fig — Additional example cell towers for both B. subtilis and E. coli. (TIF) [file pgen.1006582.s001.tif]

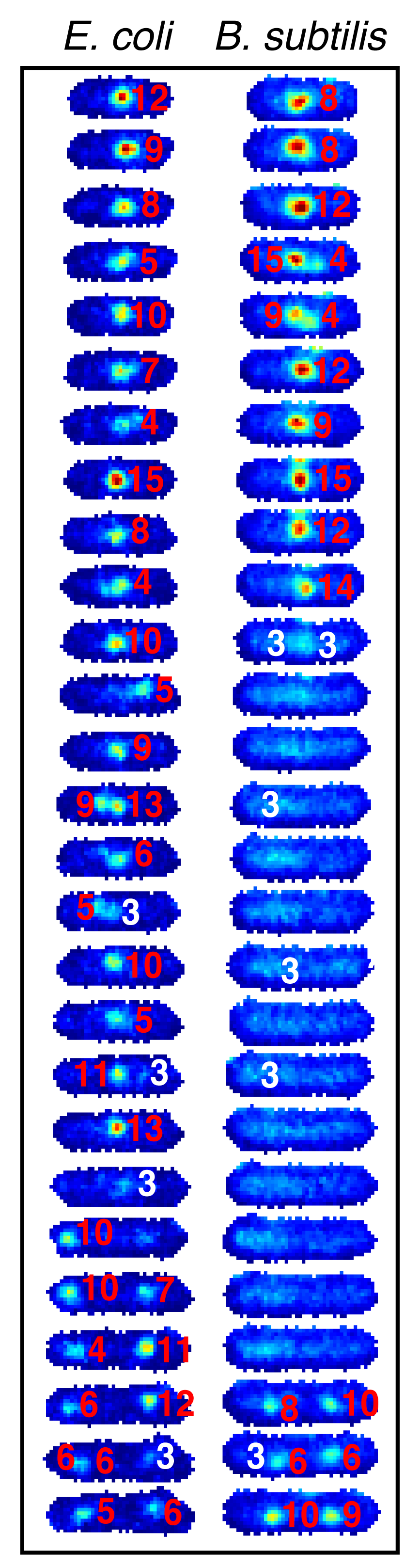

Supplement: S2 Fig — Example scored foci in partial cell towers. Frame delay is five minutes. Scores are printed in red for foci scoring high enough to be included in analysis. Foci scoring 3 or lower (white) appeared randomly throughout the cell and were excluded. (TIF) [file pgen.1006582.s002.tif]

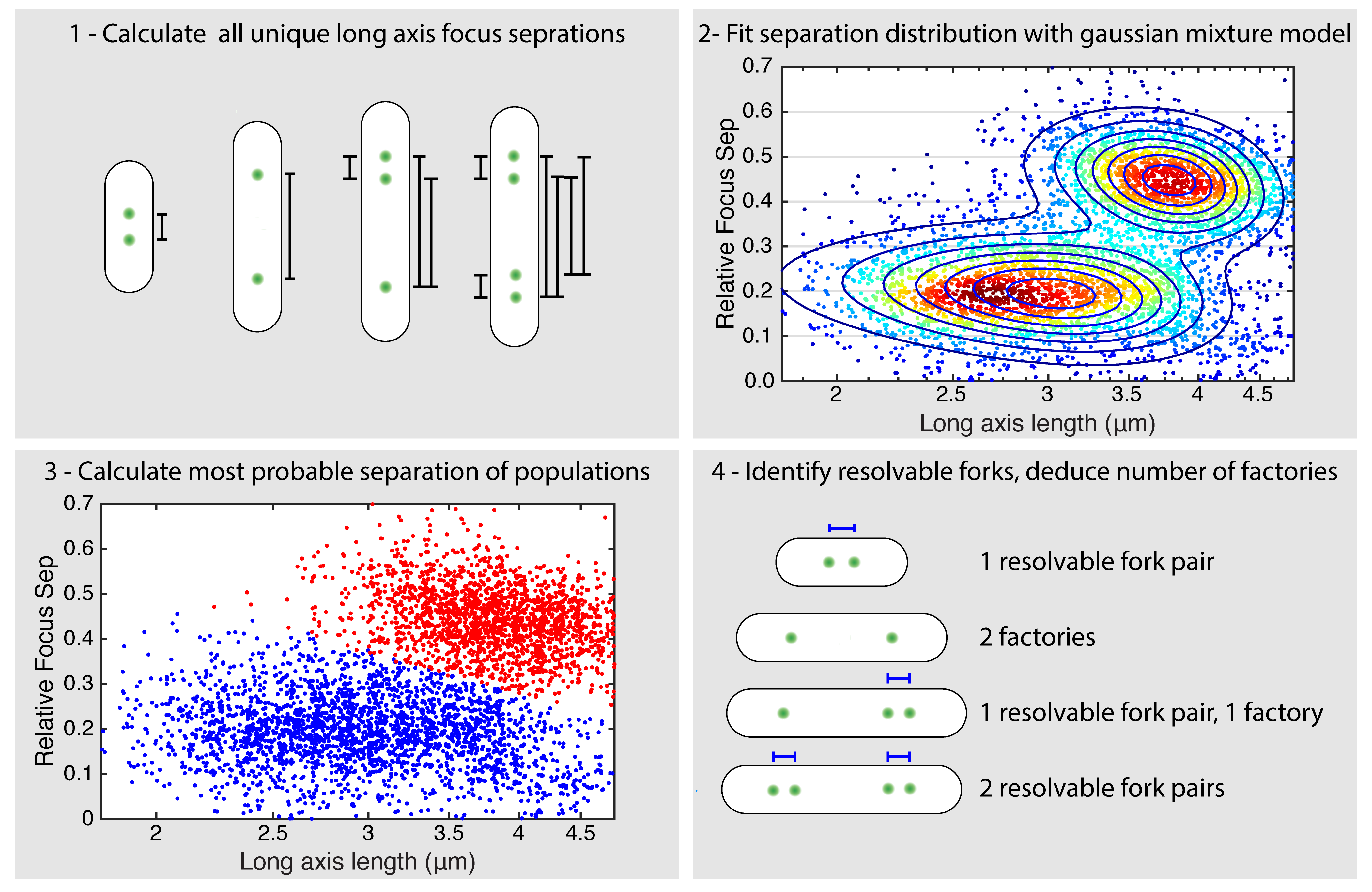

Supplement: S3 Fig — 1) All possible long axis separations are calculated (black brackets). 2) Distribution of focus separation as a function of cell length (dots) is fit with a two Gaussian mixture model (dark blue contours) using maximum likelihood. We note that the fit to the lower population is shifted slightly due to the tail, however the populations separate visually correctly. 3) Using the Gaussian mixture model obtained in step 2, each focus pair is classified as a member of the high (red) or low (blue) separation population. 4) Focus pairs that are determined to be members of the low-separation population (blue brackets) are classified as each representing an individual replisome. All other foci are inferred to be a co-localized replisome pair. (TIF) [file pgen.1006582.s003.tif]

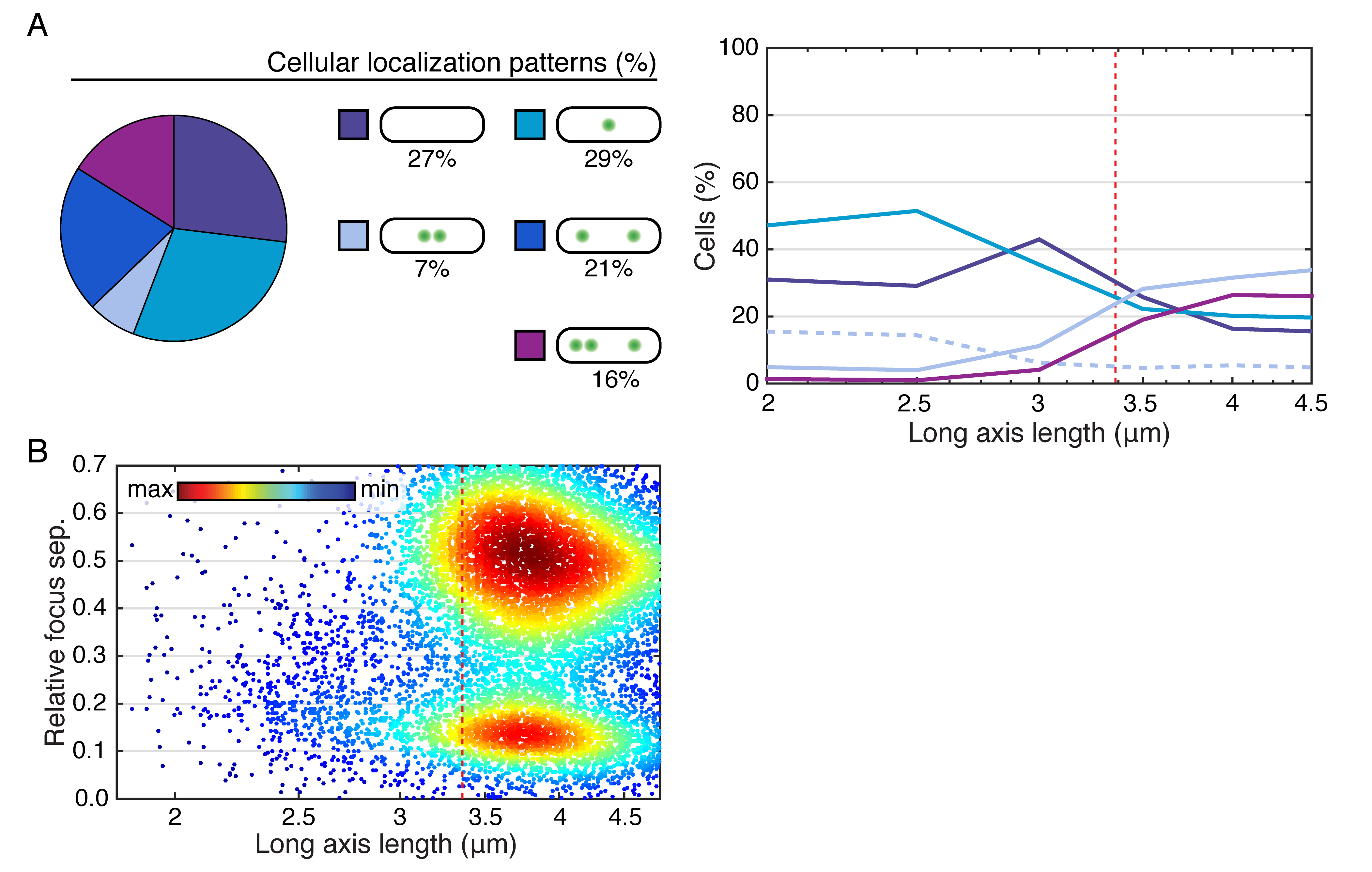

Supplement: S4 Fig — A: Relative frequencies of typical localization patterns overall (left) and separated by cell length (right). Dashed red line represents mean length at re-initiation B: Joint probability distribution for interfocus separation is used to automatically distinguish between cellular localization patterns. Dashed red line represents mean length at re-initiation. (TIF) [file pgen.1006582.s004.tif]

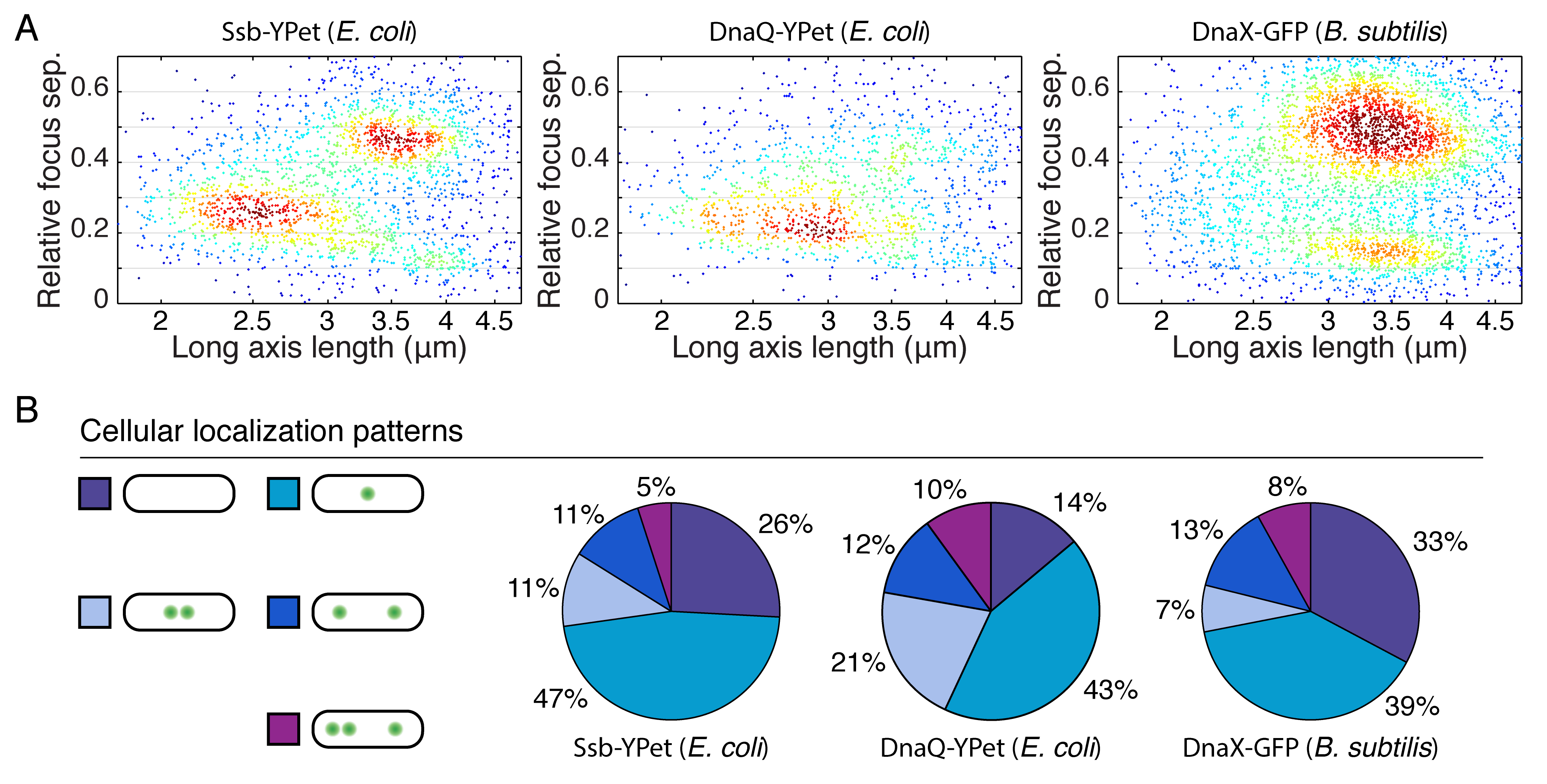

Supplement: S5 Fig — A: Joint probability distributions for interfocus separation are used to automatically distinguish between cellular localization patterns in SSB-YPet (N = 6493 Cells), DnaQ-YPet (N = 2187 cells), and DnaX-GFP (N = 10573 cells). B: Relative frequencies of typical localization patterns. (TIF) [file pgen.1006582.s005.tif]
